# The present and the future in the diagnosis and management of celiac disease

**DOI:** 10.1093/gastro/gou065

**Published:** 2014-10-17

**Authors:** Natalia E. Castillo, Thimmaiah G. Theethira, Daniel A. Leffler

**Affiliations:** Division of Gastroenterology, Beth Israel Deaconess Medical Center, Boston, MA, USA

**Keywords:** celiac disease, gluten-free diet, autoimmune diseases

## Abstract

Celiac disease is an autoimmune enteropathy caused by gluten in genetically predisposed individuals. In celiac disease, adaptive and innate immune activation results in intestinal damage and a wide range of clinical manifestations. In the past, celiac disease was thought to result in signs and symptoms solely related to the gastrointestinal tract. Now, more than half of the adult population presents with extra-intestinal manifestations that can also be expected to improve on a gluten-free diet. For this reason, it is recommended that physicians have a low threshold of suspicion for celiac disease. Current knowledge of the immune pathogenesis of this autoimmune disease has served as a catalyst for the development of novel diagnostic tools and therapeutics.

Over the years, highly sensitive and specific serological assays, in addition to genetic markers, have been found to target specific steps in the cascade pathway of celiac disease. Also the advent of the gluten challenge has enabled experts to design diagnostic algorithms and monitor clinical responses in clinical trials. The gluten challenge has provided substantial benefit in the advance of novel therapeutics as an adjuvant treatment to the gluten free diet. Generally, a strict gluten-free diet is highly burdensome to patients and can be limited in its efficacy. Alternative therapies—including gluten modification, modulation of intestinal permeability and immune response—could be central to the future treatment of celiac disease.

## Introduction

Celiac disease is defined as a chronic, immune-mediated enteropathy of the small intestine, caused by exposure to dietary gluten in genetically pre-disposed individuals [1]. Gluten is a general term for insoluble prolamine polypeptides found in wheat (gliadins and glutenins), rye (secalin), barley (hordein) and other closely-related grains [2, 3]. Unlike wheat, rye and barley, oats have been shown to be non-immunogenic in most individuals with celiac disease [4]. In susceptible individuals, gluten ingestion generates an inflammatory reaction predominantly centered in the upper part of the small intestine. Gluten-induced small intestinal mucosa injury will eventually reduce the intestinal absorptive area and interfere with the uptake of micronutrients, including fat soluble vitamins, iron, B12 and folic acid [5].

## Pathogenesis

**Figure 1.**
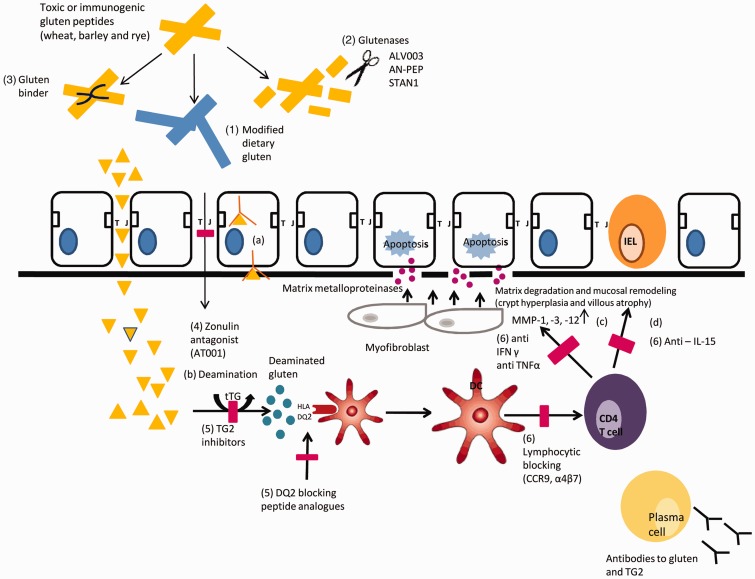
Celiac disease pathogenesis and potential novel therapeutics. Gluten peptides found in wheat, barley and rye will generate an inflammatory reaction in the small intestines of susceptible individuals. (a) Gliadin peptides will reach the *lamina propria* either by transcellular or paracellular transport mediated by zonulin. (b) De-amination by tissue transglutaminase (tTG) Type 2 increases binding affinity to human leukocyte antigen (HLA) Class II DQ2 (less common DQ8). (c) Peptide HLA-DQ complexes can induce an adaptive TH1 response that will increase cytokine production predominantly IFN-γ and matrix metalloproteinases (MMPs). The inflammatory cascade is responsible for the intestinal changes (crypt hyperplasia and atrophy of the intestinal villi) commonly seen in individuals with celiac disease. The innate immune system also contributes to the pathogenesis of celiac disease. (d) Up-regulation of interleukin-15 (IL-15) by epithelial and dendritic cells in the *lamina propria* seems to be involved in epithelial changes that are associated with refractory celiac disease Type 2 and T- cell lymphoma. Possible novel, targeted therapies are indicated in numbers. In the intestinal lumen, gluten immunogenicity can be reduced either by (i) genetically engineered grains, (ii) active proteases including ALV003, AN-PEP and STAN1 that will specifically degrade gluten into small non-immunogenic fragments and (iii) gluten binders; (iv) in the intestinal epithelium, Larazotide acetate (formerly AT-001) enhances tight junction (TJ) assembly and reduces paracellular transport of gluten to the *lamina propria*; (v) adaptive immune response may be reduced by blocking antigen presentation with transglutaminase 2 (TG2) inhibitors and HLA blocking peptides; (vi) lymphocyte blocking and anti-cytokine therapy (anti-IL-15) are other potential treatment approaches that targets TH1 activation and innate immune response.

Gluten is a glutamine- and proline-rich peptide that generates an inflammatory reaction due to its resistant nature to luminal digestion in the small intestine [1]. Intact gliadin peptides will reach the *lamina propria* by transcellular or paracellular transport, although the major mechanisms of gluten passage into the submucosa are not well characterized. Subsequent post-translation modification (de-amination) by human enzyme tissue transglutaminase Type 2 changes certain peptide-bound glutamine residues into negatively charged glutamic acid, increasing its binding affinity to human leukocyte antigen (HLA) Class II DQ2 and/or -DQ8 molecules on antigen-presenting cells (APCs) [6–8]. Peptide-HLA-DQ complexes can induce an adaptive TH1 response with a concurrent increase of interferon gamma (IFN-γ), a key cytokine in the downstream initiation of mucosal damage (Figure 1) [9].

Recent evidence has also elucidated the role of the innate immune system in the pathogenesis of celiac disease. Alterations in mRNA expression of toll-like receptor (TLR2 and TLR4) have been demonstrated in the duodenal mucosa of children with celiac disease, when compared with untreated subjects and controls [10]. Up-regulation of interleukin-15 (IL-15) by epithelial and dendritic cells in the *lamina propria* of celiac subjects seem to be involved in variations in signaling properties of intraepithelial CD8+ (Figure 1) [11]. Additionally, IL-15 increases the expression of epithelial cell surface ligands, including major histocompatibility complex Class I polypeptide-related molecule A (MIC-A), which contributes to epithelial changes and other pathological processes associated with celiac disease including refractory celiac disease Type 2 and enteropathy-associated T-cell lymphoma (EATL) [12, 13].

## Clinical features

Celiac disease is characterized by a wide range of clinical manifestations. Patients often present with gastrointestinal symptoms including altered bowel habits, abdominal discomfort, gassiness with bloating, delayed gastric emptying, and heartburn. Extra-intestinal manifestations may dominate clinical presentation in adults compare with children [8]. These prior ‘uncommon’ presentations include specific conditions like hepatopathy, dermatitis herpetiformis, IgA nephropathy, temporal lobe epilepsy, cerebellar ataxia, peripheral neuropathy, pulmonary hemosiderosis, or non-specific symptoms such as joint pain, headache and mood swings (Table 1) [14].

**Table 1.** Individuals at risk of celiac disease

**Genetic**
First-degree relatives
Trisomy syndromes
Selective IgA deficiency
**Autoimmune disorders**
Hashimoto’s thyroiditis
Type I diabetes mellitus
Dermatitis herpetiformis
Autoimmune liver disease
**Nutritional deficiency**
Refractory iron deficiency anemia
Metabolic bone disease
**Gastrointestinal disorders**
Irritable bowel syndrome
Asymptomatic elevation of aminotransferases
Malabsorption and weight Loss
**Miscellaneous**
Unexplained female infertility

## Epidemiology

Celiac disease is a commonly diagnosed autoimmune disease in areas where serology testing is available. The prevalence of celiac disease is approximately 1–2% in the populations of North and South America, North Africa, the Middle East and India [15, 16]. Reliable data is absent regarding the prevalence of celiac disease in sub-Saharan Africa and in the Asia-Pacific region [17]. In the last few decades, there has been an increase in the estimated true prevalence of celiac disease in Europe and America, consistent with the increase in other autoimmune and allergic disorders. The reasons for this increase are not clear, but are related to dietary and/or environmental changes. At the same time, the rate of diagnosis for celiac disease has been rapidly increasing. This phenomenon has been attributed to an increase in disease prevalence and the development of non-invasive diagnostic tools [18]. Lately, celiac disease has been associated with an excessive use of health care services and unnecessary therapeutic interventions prior to diagnosis [19].

It is estimated that as many as 10 million people in India and perhaps a similar number of individuals in China currently have undiagnosed celiac disease. If diagnosis rates significantly improve in either group, the number of individuals with celiac disease in these regions could surpass all of those people living with celiac disease in Europe (approximately 7 million) and in North America (approximately 5 million). This epidemic of celiac disease has the potential to result in a significant burden to healthcare resources in the Asia- Pacific region [20].

## Screening

In the post-serology era, the prevalence of celiac disease has been estimated at around 1% in the United States, yet the majority of cases of celiac disease remain undiagnosed [18]. Some experts have supported population screening for celiac disease as a cost-effective alternative that will contribute to diagnosis, but data to support this remains limited. Although celiac disease meets most of the World Health Organization (WHO) criteria for population screening for non-communicable diseases, there is little evidence to suggest a clear benefit of diagnosis and treatment in screen-detected individuals [21]. In 2004, the National Institutes of Health (NIH) Consensus Development Conference on Celiac Disease concluded that there was insufficient evidence to justify population screening for celiac disease. This intervention required further analysis that included an economic evaluation [22]. Currently, the guidelines proposed by the American College of Gastroenterology (ACCG) recommend active case-finding as the preferred strategy to increase the detection rate of celiac disease [23].

A recent randomized, controlled trial study by Kurppa *et al*. showed that treatment with the gluten-free diet (GFD) can improve histological, serological and clinical features in asymptomatic and screen-detected patients with celiac disease. Although the study sample size was relatively small and screen-detected individuals reported deterioration of social function after diagnosis, the study suggests benefit from the GFD in ‘silent cases’ and improvement in the overall health of celiac disease patients. In the future, prospective studies will be required to assess the long-term benefits of screening and treatment in preventing later complications (e.g. EATL) [24].

## Diagnostic studies

Less than 50% of adult patients currently present with classical gastrointestinal symptoms. For this reason, diagnosis requires that physicians have a high clinical suspicion for celiac disease [25]. Newly available diagnostic tools, including highly sensitive and specific serological assays and genetic markers, are far more accurate and reliable in diagnosing celiac disease than the anti-gliadin antibodies (IgA/IgG) formerly used during the 1980s and early 90s.

Currently available serological tests for the diagnosis of celiac disease are extremely accurate when compared with the other antibody-based tests used to identify other autoimmune disorders. Serological tests for celiac disease are comparable only with anti-mitochondrial and anti-thyroid autoantibody tests, which aim to evaluate primary biliary cirrhosis and autoimmune thyroiditis, respectively [15]. For this reason, serological testing should be the initial approach to assess individuals in whom celiac disease is being considered. Before serological testing for celiac disease, patients should be on a gluten-containing diet for at least a month, as serum antibodies have a half-life of 30–60 days [14]. Most commercially available antibodies, including IgA-endomysial antibody (EMA), IgA-tissue transglutaminase antibody (tTG) and IgA or IgG de-amidated gliadin peptide antibody (DGP) have a sensitivity and specificity greater than 90%. Some of the most commonly used serological tests are detailed below:

1. IgA-tTG antibody. In the late 1990s, tTG was identified as one of the antigens detected by the EMA assay, which enabled the development of high-accuracy ELISA-based tests in celiac disease. Initial assays used guinea pig antigen, resulting in higher numbers of false-positives compared with newer recombinant protein-based antigens. Currently, assays are produced by a variety of manufacturers with high accuracy rates [26]. IgA-tTG-based assays have a higher sensitivity than—and a similar specificity to—those of IgA-EMA-based testing, with lower cost [27]. IgA-tTG antibody is the preferred serological test for diagnosing celiac disease on individuals over 2 years of age [23].
2. IgA-EMA antibody. Prior to the development of IgA-tTG, IgA-EMA antibody was the preferred diagnostic tool for diagnosing celiac disease. Whilst, in a reference laboratory, EMA is still the most sensitive test, it is more technically difficult, raising concerns related to inter-observer and inter-site variability [28].
3. IgG-DGP antibody. Gluten peptides are de-amidated by intestinal tTG; resulting peptides will subsequently bind to HLA-DQ2 or DQ8 on APCs to stimulate a T-cell response [29]. The resulting antibody reaction constitutes the basis of DGP antibody testing in patients with celiac disease. IgG-DGP is more sensitive and specific than IgG-tTG and, for this reason, is the preferred test in patients with IgA deficiency [15]. In addition, DGP may be more sensitive than tTG in children under the age of 2 years.

### Special circumstances

1. Co-existing IgA deficiency. Patients with IgA deficiency have a 10–20 times greater risk of developing celiac disease [30]. Ideally, serum IgA should be initially assessed in celiac disease patients with a high pre-test prevalence. In patients with low IgA levels, IgG-based DGP and/or tTG testing should be considered to be part of the serological assessment [23]. A recent study has reported that IgG anti-tTG was more specific—although less sensitive—for celiac disease than IgG anti-DGP [31].
2. False positive results. Despite having high sensitivity and specificity rates, a positive serological test does not confirm the diagnosis of celiac disease. In most individuals, IgA-tTG antibody testing has a very high negative predictive value. In rare circumstances, IgA-tTG can yield a false-positive result due to cross-reaction of antibodies. Some conditions that can render a false-positive result are—but are not limited to—an enteric infection, congestive heart failure, chronic liver disease and hypergammaglobulinemia [15].
3. False negative results. The most common reason for a ‘false negative’ tTG is that the patient is already on a low-gluten diet. IgA-tTG antibody testing is less sensitive in younger children (under 2 years) because the immune system is immature. As previously described, primary IgA deficiency is another medical condition that could potentially show a negative IgA based testing [15].

Point-of-care tests (POTC) based on transglutaminase 2 (TG2) auto-antibodies are increasingly being marketed as a replacement for serum-based testing [14]. Evidence for and against POCT is still in a nascent stage [32, 33]. Despite the skepticism displayed by clinicians towards these ‘off-the-shelf’ tests, POCT kits may have a key role in increasing disease detection rates in countries with limited health resources and large numbers of potential celiac subjects.

## Genetic markers

The most important recognized genetic risk factor for celiac disease is the presence of HLA-DQ2 or DQ8, of which one or both will be present in virtually all patients with celiac disease [7]. Most of the remaining celiac disease population (less than 1%) will carry half of the HLA-DQ heterodimer [34]. HLA-DQ2 and DQ8 genetic testing have a very high negative predictive value (more than 99%). This feature has been proven to be useful in ruling out celiac disease in patients with equivocal duodenal biopsies, or in those who are already following a GFD and are reluctant to have a gluten challenge [35]. The utility of HLA testing has also been critical in differentiating non-celiac gluten sensitivity from celiac disease [36]. Recent genome-wide association studies [GWAS] have identified many novel non-HLA loci that have partially explained some of the genetic variants in celiac disease. The existence of a large number of non-HLA genes, partly shared by each individual patient, suggests that celiac disease may be more heterogeneous than previously considered [37].

## Gluten challenge

Celiac disease is a unique autoimmune disorder in which a GFD is the only effective and accepted treatment. The GFD resolves celiac-related immune dysregulation characterized by abnormal serological titers and mucosal injury. Patients with celiac disease who are on a GFD prior to diagnostic testing will usually yield negative serologies and normal duodenal histologies [38]. The gluten challenge is clinically relevant in patients with suspected but unproven celiac disease that has been previously treated with a GFD. Its aim is to return to a normal, gluten-rich diet under medical supervision that will enable diagnostic testing [39]. This test is not suitable for individuals with suspected celiac disease who experience severe symptoms or neurological manifestations after gluten ingestion.

The gluten challenge remains the ‘gold standard’ for diagnosis of celiac disease in positive HLA patients on a GFD. Previously, the gluten challenge involved consuming at least 10 g of gluten per day for a 6–8 week period. A recent study has demonstrated that one to two servings (> 3 g) of gluten daily for two weeks, followed by serological testing and duodenal biopsy, are sufficient to induce histological and serological changes in the majority of individuals with celiac disease [23, 39]. Its diagnosis can be definitely excluded if serological and duodenal biopsy results are normal following the 6–8 weeks of the gluten challenge. However, this approach can be troublesome and many patients are unwilling to undergo classical- or even the modified gluten challenge, due to exacerbation of symptoms. For this reason, novel techniques have been explored, including *in vitro* exposure of duodenal biopsy specimens to gluten. Tortora *et al*. found that *in vitro* gliadin-induced HLA-DR expression is an accurate tool for the diagnosis of celiac disease [40]. A recent study by Vanga *et al*. analysed the difference in cytokine release in gluten-stimulated-, compared with non-stimulated, biopsies between celiac disease subjects and healthy controls. The study showed a significant increase in several cytokines including TNF-α, IFN-γ, IL-6 and IL-10: all crucial in the pathogenesis of celiac disease. Moreover, a score based on the increments of three of these cytokines (TNF-α, IFN-γ and IL-10) appeared to provide a 100% diagnostic accuracy in differentiating healthy controls from treated celiac disease patients with normal duodenal histology [41]; however, none of these tests are clinically available at this time.

The gluten challenge has also supported the development of other reliable diagnostic tools. Intestinal fatty acid binding protein (I-FABP) is a cytosolic protein that is release by necrotic enterocytes. I-FABP has previously been studied as a possible marker to evaluate mucosal damage. This protein has been proposed as a sensitive diagnostic test in the evaluation of ischemia in mechanical small bowel obstruction [42]. On the other hand, I-FABP has demonstrated a positive predictive value of 98% in children with a positive serological test for celiac disease [43]. I-FABP levels increased significantly after two weeks of gluten challenge, while lower levels of I-FABP were seen in individuals following two weeks on gluten withdrawal. I-FABP may be considered as a future diagnostic tool that could assist in the diagnosis of celiac disease.

## Upper endoscopy and small-bowel biopsy

Despite the development of highly sensitive serological assays, small-bowel mucosal biopsy is still considered the ‘gold standard’ for diagnosis of celiac disease. The US NIH consensus statement suggests serological testing as the first step in evaluating celiac disease. Duodenal biopsy is strongly recommended in individuals with clinical symptoms suggestive of celiac disease, subjects with a positive celiac antibody test result, and in cases where serological results are non-diagnostic [44]. Positive serological results, along with typical celiac disease biopsy findings (intra-epithelial lymphocytosis, crypt hyperplasia, and villous atrophy) are highly suggestive of celiac disease. A repeat biopsy while on the GFD is no longer required for a definitive diagnosis of celiac disease, but may be useful in follow-up [38]. In view of the patchy nature of celiac disease, it is suggested that at least four to six endoscopic biopsy specimens be taken from the duodenum, with two samples from the bulb region. Biopsies from the duodenal bulb should be carefully interpreted, since other conditions may present with similar histological findings to those seen in celiac disease [38].

### Special circumstances

1. Duodenal lymphocytosis with normal villous architecture. This histological finding can be found either in individuals with partially treated celiac disease or several other conditions that include—but are not limited to—*helicobacter pylori* infection, bacterial overgrowth, non-steroidal anti-inflammatory drugs use, inflammatory bowel disease (IBD), tropical sprue, and lymphocytic enteritis [45].
2. Villous atrophy with negative serology. This finding represents a diagnostic dilemma. A retrospective study by DeGaetani *et al*. showed that sero-negative celiac disease, medication-related villous atrophy and unclassified sprue were the most common differential diagnoses among 72 patients with villous atrophy, assessed over a 10-year period [46, 47].

**Figure 2.**
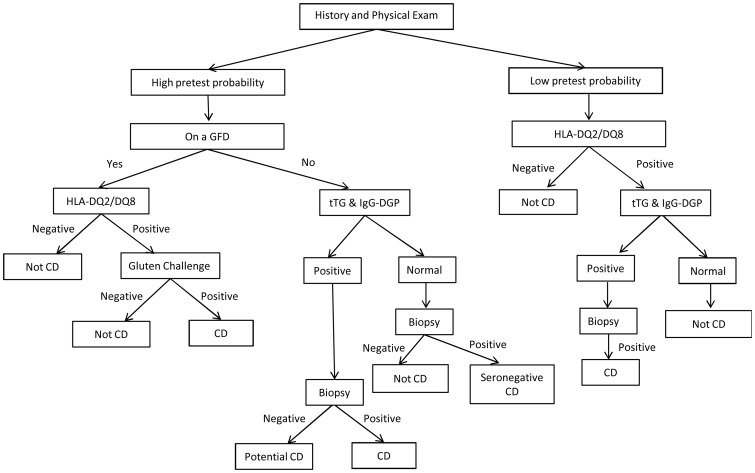
Proposed algorithm for celiac disease diagnosis. GFD = gluten free diet; HLA = human leukocyte antigen; CD = celiac disease; DGP = de-amidated gliadin peptide; tTG = tissue transglutaminase.

The development of highly sensitive and specific serological assays, in addition to genetic markers and novel diagnostic tools, has positively influenced current diagnostic algorithms (Figure 2). The recent European Society for Paediatric Gastroenterology, Hepatology and Nutrition (ESPGHAN) guidelines have proposed serological evaluation as an alternative to invasive techniques, such as endoscopically guided biopsies, in the pediatric population, but further evidence is still required to support this approach in the general population.

## Treatment: now and then

Interestingly, current knowledge of the gluten-free diet as the only available and accepted treatment for celiac disease dates back to the 1940s, following the recognition of gluten as the main culprit of the disease [48].

### The gluten-free diet: a less engaging option

Both genetic and environmental factors contribute to the pathogenesis of celiac disease [49]. The dramatic improvement in sanitation, along with increased consumption of refined grains in the human diet as part of the so called ‘agricultural revolution’, may be partially responsible for the rise in celiac disease and other food intolerances [50]. The gluten-free diet is the only available and accepted treatment. Complete elimination of wheat, barley and rye from the diet is not feasible and impractical, thus a strict gluten-free diet essentially refers to a high level of gluten restriction, such that levels consumed are safe for most individuals [23]. The exact amount of gluten that people can tolerate without developing deleterious effects is difficult to assess and probably varies between individuals. In one study, most patients could tolerate up to 50 mg of gluten per day, equivalent to 500 g of food containing 20 ppm of gluten, but a recent systematic review considered less than 10 mg of daily gluten intake to be safe and unlikely to cause significant abnormalities [51, 52].

Gluten threshold variability within the population may largely contribute to ongoing clinical symptoms and worsening histological changes. Actually, 10–30% of patients with celiac disease will have persistent symptoms, signs or laboratory abnormalities, despite being on a gluten-free diet for 12 months, and would be classified as non-responders [53]. Non-responsive celiac disease (NRCD) is largely caused by inadvertent exposure to gluten that accounts for 35–50% of persistent symptoms in patients with celiac disease [23]. Other etiologies, such as lactose and fructose intolerance, small intestine bacterial overgrowth (SIBO) and irritable bowel syndrome (IBS) should be considered in the differential diagnosis of NRCD.

In general, dietary regimens or modifications are the least appealing strategies of all medical modalities available to counteract any given pathology [54]. Long-term adherence to a gluten-free diet is estimated to range between 17–45% of adults [55]. The challenge of managing a lifelong gluten-free diet arises from the restrictive nature of the diet that will force most of patients with celiac disease to bring their own food while traveling and to avoid eating out [56]. Poor product availability, difficulties over labeling and high costs within the large existing gluten-free market further contribute to dissatisfaction among patients and negative health outcomes [57]

Treatment based solely on a strict gluten-free diet is highly burdensome to patients and has its limitations in efficacy. Advances in the understanding of the immunopathogenesis of celiac disease have not only supported novel diagnostics, but have also contributed to the development of alternative novel therapeutic strategies that could improve a person’s overall health and quality of life. For this reason, three main approaches have been proposed as new therapeutic modalities that include: gluten detoxification, inhibition of intestinal permeability, and modulation of immune response (Table 2).

**Table 2.** Summary of the main therapeutic approaches for celiac disease.

Target	Aim	Therapeutic modalities/mechanisms	Compound	Development stage (clinical trial)
Toxic or immunogenic gluten peptides (wheat, barley and rye)	Dietary modification	Gluten-free diet		Only available and accepted treatment
	Reduction of gluten immunogenicity	Genetically engineered grains		
		Active proteases	ALV-003	Phase II
		Gluten binders	Copolymer P(HEMA-co-SS)	Pre-clinical
		Probiotic preparation	Lactobacilli	Phase I
Intestinal epithelial barrier	Intestinal permeability modulation	Tight junction regulation	Larazotide acetate (formerly AT-001)	Phase II
Autoimmune response	Modulation of overactive immune response	Induction of immune tolerance	Nexvax 2	Phase I
		Reduction of pro-inflammatory TH1 cell and regulatory T-cell responses	*Necator Americanus*	Phase Ia/IIb
Inflammatory response	Reduction of cytokine production	Monoclonal antibodies against TNF- α	Infliximab*	Phase I
		Humanized IL-15- specific antibody	Hu-MiK-Beta-1	Phase I
Innate immune response	Lymphocyte recruitment blockade	Anti-CCR9 blockade	CCX282-B agent	Phase I

*Infliximab has been anecdotally successful in steroid-refractory celiac disease

### Gluten detoxification

Western diet is mainly based in gluten-containing grains that are highly toxic and immunogenic for patients with celiac disease. Safe alternatives, such as oats, have been considered, but these grains are not uniformly recommended and may lack relevant dietary nutrients [58]. Genetically engineered grains have been studied as a second option to reduce gluten toxicity. Wheat strains with a low content of α and β gluten may reduce immunogenicity, while other gluten epitopes could enhance immune response. It has been shown that the genetic modification by deletion of α gliadin locus from the wheat genome could reduce T-cell stimulatory epitopes [59, 60]. Nonetheless, wheat plants have several hundred genes encoding for harmful gluten epitopes that are difficult to target.

On the other hand, active proteases specifically degrade gluten into small non-immunogenic fragments before they can transit across the small intestinal mucosa. ALV003 is an active oral protease that combines two enzymes—cysteine endoprotease B-isoform 2 (EP-B2 or ALV001) and prolyl endopeptidase (SC PEP or ALV002)—derived from barley and bacteria, respectively. Together, these degrade gluten more effectively than either enzyme alone [61]. Tye-Din *et al*. demonstrated *in vivo* that ALV003 could abolish peripheral blood T-cell IFN-γ responses induced by the administration of gluten over a 3-day period in patients with celiac disease [62].

Additional clinical trials have been conducted to test the safety and tolerability of single doses of ALV003 and to determine the gastric pharmacokinetic and pharmacodynamics profiles, both in a fasted stated and after a gluten-containing meal. In the first two-phase I single-blind, placebo-controlled cross-over trial in healthy subjects and in patients with celiac disease, ALV003 appeared to be well tolerated up to the maximum dose (1800 mg) with no related serious or severe adverse events. ALV003 was able to degrade gluten in the human stomach in a manner comparable to previous *in vitro* data, remaining stable and active in an acidic gastric environment [63].

Similar results regarding safety and tolerability have been reported in phase II clinical trials, where ALV003 attenuates gluten-induced small intestinal mucosal injury in patients with celiac disease after a 6-week gluten challenge. This is very promising; however, the impact of glutenases in other dietary proteins, alternative routes for delivery of glutenases, and the exact amount of gluten detoxified *in vivo* that would enable patients either to consume large quantities of gluten or merely avert intestinal inflammation, are key elements to be considered in the advancement of glutenases in clinical trials.

Alternative therapies at pre-clinical stage, including probiotic preparations by lactobacilli and further glutenase therapy at the early clinical stage such as AN-PEP and other commercially-available food-grade enzymes (STAN1), could constitute further safe and well-tolerated strategies for detoxification of dietary gluten [64–66].

### Intestinal permeability modulation

The intestinal epithelium constitutes the main barrier to endogenous and exogenous stimuli. In a physiological state, immunogenic antigens can cross the mucosal barrier by two pathways: transcellular and paracellular, the latter being involved in tight junction regulation [67]. Recent insights into the intricate mechanism that regulates intestinal epithelial paracellular pathways have led to the discovery of zonulin, a protein widely investigated in a variety of clinical conditions including celiac disease [68].

Larazotide acetate (formerly AT-001) is an 8-mer peptide and tight junction (TG) regulator that controls cellular changes induced by gliadin and cytokines. As shown in *in vitro* studies across Caco-2 cell monolayers, larazotide inhibits gliadin 13-mer peptide translocation, a peptide highly implicated in celiac disease [69, 70]. Conversely, *in vivo* studies have demonstrated inhibition of gliadin-induced macrophage accumulation in the small intestine and preservation of TG structure [71].

Three phase II human clinical trials have made larazotide a suitable and potential candidate for treating celiac disease. All randomized, placebo-controlled trials have appeared to be safe, well tolerated and effective in reducing gastrointestinal symptoms after a gluten challenge [72–74]. Preliminary results from the most recent phase II b trial of larazotide acetate have shown substantial clinical improvement in both gastrointestinal and non-gastrointestinal symptoms in patients with celiac disease on a GFD lasting ≥12 months at the lowest dose (0.5 mg), as compare with placebo (Clinical Trials registration number NCT01396213). Growing supporting data on the safety and efficacy of larazotide acetate suggests that this may be a promising agent for the treatment of celiac disease.

### Modulation of immune response

The recent increase in celiac and other inflammatory diseases is attributed not only to genetic predisposition; rather, environmental factors clearly play a role in the regulation of immune response [75]. Recent changes in health practices, such as vaccination, and the absence of ‘old friends’ or microbes that were once abundant, have been further studied as possible therapeutic alternatives that could restore immune tolerance in celiac disease [76].

The induction of tolerance through vaccination has been widely considered in allergic-, other autoimmune- and celiac diseases. Nexvax 2, a gluten-specific therapeutic vaccine, is a combination of three peptides (gliadin, hordein and secalin) commonly identified by T-cells in HLA-DQ2 genotype patients, that will ultimately reprogram gluten-specific T-cells. A phase I randomized clinical trial has shown Nexvax 2 to be safe and well tolerated after weekly injections over 3 weeks in celiac disease patients on a GFD (Clinical Trials registration number NCT00879749). Nexvax 2 appeared to induce a biological response, and further research is expected on the efficacy of vaccination in restoring immune tolerance to gluten.

Epidemiological data, experimental models and therapeutic trials in other inflammatory diseases, such as inflammatory bowel disease (IBD), have successfully used pig whipworm (*trichuris suis*) as an alternative therapy with the hope of reducing pro-inflammatory Th1 cell and regulatory T-cell responses [77]. Conversely, the benefits of gastrointestinal nematode (*necator Americanus*) have been explored in a phase Ib/IIa study in patients with celiac disease following a 5-day, high-dose gluten challenge. This study failed to show significant effect on duodenal histology Marsh scores, but enhanced response of IL-1B and IL-22 and decreased levels of IFN-γ and IL-17 levels, both relevant cytokines in the pathogenesis of celiac disease.

In celiac disease, the pro-inflammatory response along the immunogenic pathway results in the production of cytokines, such as TNF-α, IFN-γ and IL-15, suggesting a plausible approach with monoclonal antibody-based therapies. Monoclonal antibodies against TNF-α (infliximab) have been extensively studied in inflammatory bowel disease while, anecdotally, infliximab has been successful in the treatment of some cases of refractory celiac disease [78]. Moreover, humanized IL-15-specific antibody is a promising therapy to treat Type 2 refractory celiac disease and EATL. Monoclonal antibodies to IL-15 effectively target and block the signaling pathway by which IL-15 transmits anti-apoptotic signals (Jak3/STAT5) to NK and CD8+ T lymphocytes [13]. A phase I clinical trial, currently under way, will assess the safety and efficacy of the humanized Hu-MiK-Beta-1 monoclonal antibody in patients with refractory celiac disease, as an alternative treatment beyond supportive measures and steroids (Clinical Trials registration number NCT01893775).

Other autoimmune diseases, such as rheumatoid arthritis, diabetes Type 1 and IBD have largely contributed to the development of further possible therapies for the treatment of celiac disease. Less well-known and effective therapies include celiac-specific HLA inhibition, CCR-9 blockade by CCX282-B agent and lymphocyte recruitment blockade: most of these treatments have been extensively studied in connection with Crohn’s disease and appeared to be effective. CCX282-B is the only agent that has been evaluated in a phase II clinical trial of small-intestinal biopsies from patients with celiac disease before and after gluten exposure. No unexpected serious events occurred and no worrying changes in laboratory parameters were seen (Clinical Trials registration number NCT00540657).

## Conclusion

Celiac disease is a unique autoimmune disorder. Despite being well characterized, a majority of patients are still under-diagnosed. The knowledge of gluten as a trigger peptide in the immune pathogenesis of celiac disease has driven the development of non-invasive diagnostic tools and alternative therapies. In the post-serology era, the gluten challenge has shown itself to be beneficial in the design of clinical trials. In more recent years, novel therapies have shown the potential to be effective and safe, but true efficacy and long-term benefits are still uncertain. Further research will be required to further improve diagnosis and management of celiac disease.

## Funding

Consulting or grants from the following: Prometheus diagnostics, Alba pharmaceuticals, Alvine therapeutics, Shire therapeutics to D. L.

*Conflict of interest statement*. none declared.
